# Beyond the Clinic Walls: Examining Radiology Technicians’ Experiences in Home-Based Radiography

**DOI:** 10.3390/healthcare12070732

**Published:** 2024-03-27

**Authors:** Graziano Lepri, Francesco Oddi, Rosario Alfio Gulino, Daniele Giansanti

**Affiliations:** 1 Azienda Unità Sanitaria Locale Umbria 1, Via Guerriero Guerra 21, 06127 Perugia, Italy; graziano.lepri@uslumbria1.it; 2 Facoltà di Ingegneria, Università di Tor Vergata, Via del Politecnico, 1, 00133 Rome, Italy; francesco.oddi@alumni.uniroma2.eu (F.O.); gulino@disp.uniroma2.it (R.A.G.); 3 Centro Nazionale TISP, Istituto Superiore di Sanità, Viale Regina Elena 299, 00161 Rome, Italy

**Keywords:** radiology, home radiology, CAWI, technology assessment

## Abstract

In recent years, the landscape of diagnostic imaging has undergone a significant transformation with the emergence of home radiology, challenging the traditional paradigm. This shift, bringing diagnostic imaging directly to patients, has gained momentum and has been further accelerated by the global COVID-19 pandemic, highlighting the increasing importance and convenience of decentralized healthcare services. This study aims to offer a nuanced understanding of the attitudes and experiences influencing the integration of in-home radiography into contemporary healthcare practices. The research methodology involves a survey administered through Computer-Aided Web Interviewing (CAWI) tools, enabling real-time engagement with a diverse cohort of medical radiology technicians in the health domain. A second CAWI tool is submitted to experts to assess their feedback on the methodology. The survey explores key themes, including perceived advantages and challenges associated with domiciliary imaging, its impact on patient care, and the technological intricacies specific to conducting radiologic procedures outside the conventional clinical environment. Findings from a sample of 26 medical radiology technicians (drawn from a larger pool of 186 respondents) highlight a spectrum of opinions and constructive feedback. Enthusiasm is evident for the potential of domiciliary imaging to enhance patient convenience and provide a more patient-centric approach to healthcare. Simultaneously, this study suggests areas of intervention to improve the diffusion of home-based radiology. The methodology based on CAWI tools proves instrumental in the efficiency and depth of data collection, as evaluated by 16 experts from diverse professional backgrounds. The dynamic and responsive nature of this approach allows for a more allocated exploration of technicians’ opinions, contributing to a comprehensive understanding of the evolving landscape of medical imaging services. Emphasis is placed on the need for national and international initiatives in the field, supported by scientific societies, to further explore the evolving landscape of teleradiology and the integration of artificial intelligence in radiology. This study encourages expansion involving other key figures in this practice, including, naturally, medical radiologists, general practitioners, medical physicists, and other stakeholders.

## 1. Introduction

### 1.1. Background

Within the transformative realm of domiciliary radiology, illuminated by the exhaustive scoping review led by Toppemberg et al. [1], a profound shift in healthcare delivery is discernible. Spanning from the pioneering initiatives of Losev in 1958 [2] to contemporary endeavors exemplified by Mark et al.’s establishment of a domiciliary-based X-ray response team in 2022 [3], a palpable evolution toward a patient-centric ethos in radiological practices unfolds. While an array of studies illuminates promising outcomes, encompassing noteworthy cost-effectiveness [4], intricate operational dynamics [5], and a resounding positive reception from patients [6], a compelling necessity emerges for a meticulous exploration into the experiential landscape of professionals within this dynamically evolving field.

The landmark survey conducted by Sawyer et al. in 1995 [7], resonating with the unanimous acknowledgment among practitioners regarding the paramount significance of domiciliary radiography, triggers a critical examination of the nuanced challenges encountered by these professionals. Recent inquiries led by Andersen et al. [5] and Dollard et al. [6], offering invaluable insights into the operational nuances and patient perspectives, further illuminate the multifaceted nature of assimilating radiological services into the fabric of non-clinical settings. While economic analyses by Kjelle et al. [4] present commendable evidence of cost reduction, Aldridge et al.’s scrupulous study [8] underscores the need for qualitative investigations to glean a comprehensive understanding. The exhaustive analysis conducted by Kjelle and Lysdahl [9], reaffirming the potential advantages of domiciliary radiology, accentuates reductions in hospital transfers and the assurance of timely diagnoses. In a broader societal context, public–private partnerships, as exemplified by Datta et al. (2017) [10], illustrate the potential impact of collaborative efforts in addressing healthcare gaps. The success of this specific initiative in detecting pulmonary TB highlights the broader role such partnerships can play in scaling up and designing impactful interventions. This small sample of recent studies (although a specific review study would undoubtedly provide an even broader perspective) already serves as an illustration of how domiciliary radiology can be conducted in various locations and settings, each with a different focus, as outlined in Table 1.

In navigating these intricately woven dimensions, it becomes imperatively clear that a comprehensive technology assessment is not merely a desirable but an essential undertaking. The narrative, gracefully meandering through historical foundations, the intertwined perspectives of practitioners and patients, the intricacies of operational challenges, economic considerations, and collaborative models, resoundingly underscores the transformative potential embedded within domiciliary radiology. This evocative landscape underscores the need for a meticulous investigation into the experiences and perspectives of professionals operating within this evolving field, as has been conducted in other fields of digital radiology in several applications, including teleradiology and the integration of artificial intelligence [11,12,13,14,15,16,17,18,19,20,21,22,23,24,25,26,27]. An overview of surveys provides a nuanced understanding of various aspects of the field. Starting with a snapshot of teleradiology practice in Turkey, Dicle et al. delve into the practicalities and challenges faced by radiologists [11]. Transitioning to Ghana, Dzefi-Tettey et al. explore the perceptions of clinical medical students regarding a career in radiology, shedding light on the factors influencing future professionals in the field [12]. Vabo et al.’s survey focuses on patient-reported outcomes after fracture treatment in primary healthcare, providing insights into the impact of initial conservative approaches [13]. On the technological front, Macedo et al. evaluate the usability and efficiency of an application in orthopedics, emphasizing the integration of technology into diagnostic processes [14]. The socio-economic and psychological repercussions of the COVID-19 outbreak on radiologists are investigated by Florin et al., offering a glimpse into the challenges faced by practitioners [15]. In Japan, Yamashiro et al. present survey results on work-style reform and technology utilization among diagnostic radiologists, reflecting the evolving landscape of radiological practices [16]. A comprehensive survey encompassing radiologists, medical students, and surgeons by van Hoek et al. underscores skepticism about artificial intelligence and the potential evolution of the radiology field [17]. Turning to the realm of teleradiology, Coppola et al. present Italian survey results, while Jacobs et al. explore patient satisfaction with teleradiology services in general practice [18,19]. The on-call service of neurosurgeons in Germany is investigated by Brenke et al., revealing organizational aspects and the acceptance of modern technologies [20]. Meanwhile, Kim et al. gauge the attitude of Korean primary care family physicians toward telehealth, offering insights into the acceptance and perspectives of telehealth services [21]. Examining factors influencing clinician satisfaction with radiology services, Lindsay et al. contribute to the discourse on service quality [22]. Winblad et al.’s nationwide survey in Finland sheds light on the positive aspects found in healthcare information and communication technology implementation [23]. Finally, Ninos et al. focus on the development and evaluation of a PDA-based teleradiology terminal, emphasizing advancements in technology and diagnostic capabilities [24]. CAWI tools could be a valid aid, as demonstrated under the COVID-19 pandemic [25] and in the investigation of the acceptance of the integration with artificial intelligence [26,27]. Collectively, these surveys weave a narrative that encompasses technological advancements, practitioner perspectives, patient outcomes, and the evolving landscape of radiological practices. The discourse not only underscores the current state of the field but also hints at potential future directions, emphasizing the need for continuous adaptation and innovation in the dynamic field of radiology. The application of surveys in home/domiciliary radiology could provide a nuanced understanding of various aspects of this specialized field. Such an exploration is not merely an academic endeavor but a crucial undertaking to comprehend the intricate challenges, gain unique insights, and consider the pragmatic aspects confronted by these professionals. A dedicated investigation into their experiences could not only enhance our understanding but also shape strategies and policies aligned with the dynamic nuances of domiciliary radiology. This, in turn, contributes to fostering its seamless integration into contemporary healthcare practices. Overall, the brief literature analysis highlights the need for targeted surveys among professionals directly involved in home radiology practice to gather valuable and structured information for enhancing and promoting this approach. From a healthcare perspective, this practice can bring numerous advantages, as seen in this brief review, by shifting the practice to the patient’s home and avoiding complex hospital visits. Fragile and/or significantly disabled patients, for instance, can benefit significantly from the spread of home radiology. The healthcare system can also gain several advantages, as it prevents potential risks of worsening for these patient categories.

### 1.2. The Rationale for the Study and Purpose

Exploring home radiology involves addressing pivotal questions spanning logistical, training, patient care, and technological aspects. Key inquiries include optimizing logistical challenges, defining essential skills for technicians, assessing patient care impact, understanding technological requirements, implementing quality control, gauging technician opinions, tracking industry evolution, leveraging patient feedback, and identifying specific populations or scenarios where domiciliary imaging excels or faces challenges. This comprehensive framework sheds light on the inherent opportunities and obstacles in the dynamic field of home radiology.

The aim of the study is to conduct a *pilot study* facing a comprehensive investigation into home radiology by scrutinizing the experiences, challenges, and perceptions of medical radiology technicians, with the overarching goal of informing strategies for the optimal integration of domiciliary radiology into modern healthcare practices.

## 2. Methods

The research methodology hinged upon the deployment of a comprehensive questionnaire facilitated by a cutting-edge CAWI tool. This instrument was strategically disseminated not only to citizens but also to other professionals potentially engaged in the realm of home radiology practices in the *health domain*. 

The participants in the pilot study were contacted using peer-to-peer methods, which leveraged messenger/chatting groups and social media platforms. These methods were used to select participants based on professions and on their affiliations with professional associations. Throughout this outreach process, utmost care was taken both to ensure the privacy of the participants was respected during all interactions and to reach the entire national territory.

To facilitate the data-collection process, Computer-Assisted Web Interviewing (CAWI) tools were employed. These tools were customized with different menus and sets of questions, which were tailored to the specific professions declared by the participants in the initial survey questions. This customization ensured that the questions were relevant and appropriate for each participant group. The development of the CAWI tools was executed utilizing Microsoft Forms, a deliberate choice owing to its seamless integration with the Office 365 (Version 2024) suite provided to the Tor Vergata University staff. Notably, Microsoft Forms boasts certification for compliance with prevailing IT security regulations from a systems perspective.

This choice was, therefore, influenced by the tool’s integration within the university’s Office 365 suite and its official approval for research purposes. Selecting an alternative external tool would have necessitated additional approval processes, which were not guaranteed and would have entailed a significant expenditure of time and resources.

Overall, these strategic decisions regarding participant outreach and data collection tools were made to ensure the efficiency, reliability, and ethical integrity of the research process.

Within the confines of this pilot study, our analytical focus has been steadfastly directed toward scrutinizing the outcomes derived from the detected perspectives of medical radiology technicians (MRTs). As the linchpin figures in the delivery of home radiology practices, their insights carry paramount significance. It is pertinent to note that our ongoing efforts extend beyond this specific cohort, encompassing a broader spectrum of stakeholders. Furthermore, we introduced a secondary CAWI tool tailored for experts affiliated with national scientific societies and the national associations of professionals integral to this phase of the project. 

The dissemination of both CAWI instruments occurred in a peer-to-peer fashion, ensuring anonymity, and leveraged social networks and other channels affiliated with the scientific societies and associations involved. This approach was meticulously crafted to uphold the utmost standards of privacy and confidentiality. The following modules were used in the CAWI:

- Single choice questions;
- Multiple choice questions;
- Evaluation (graded) questions (with a 6-level psychometric scale);
- Likert questions with a 6-level scale;
- Open-ended questions (in a few cases).

The *principal CAWI tool is the electronic survey (ES)*, which allows the collection of feedback from the actors related to the home radiology practice. 

**Figure 1:**
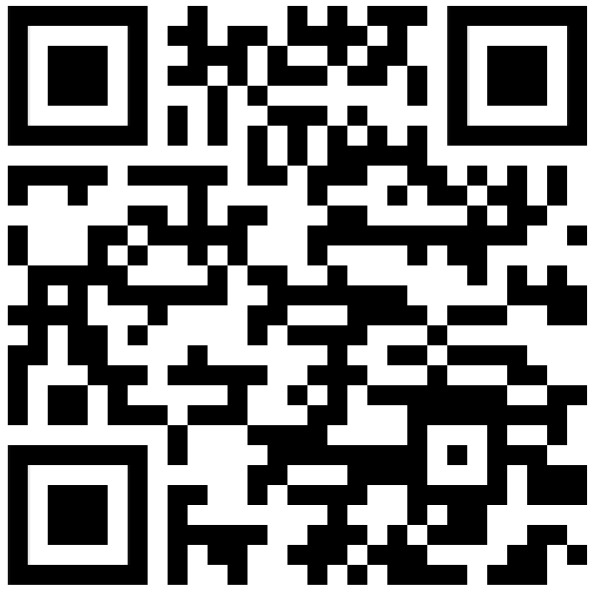
The QR code of ES.

The link and the QR code for the electronic survey are as follows: https://forms.office.com/e/fW1w6YbwNr (accessed on 15 March 2024) (see Figure 1).

The *second tool*, the CAWI tool, is the *electronic feedback form* (EFF) dedicated to the experts of the scientific societies/scientific associations.

**Figure 2:**
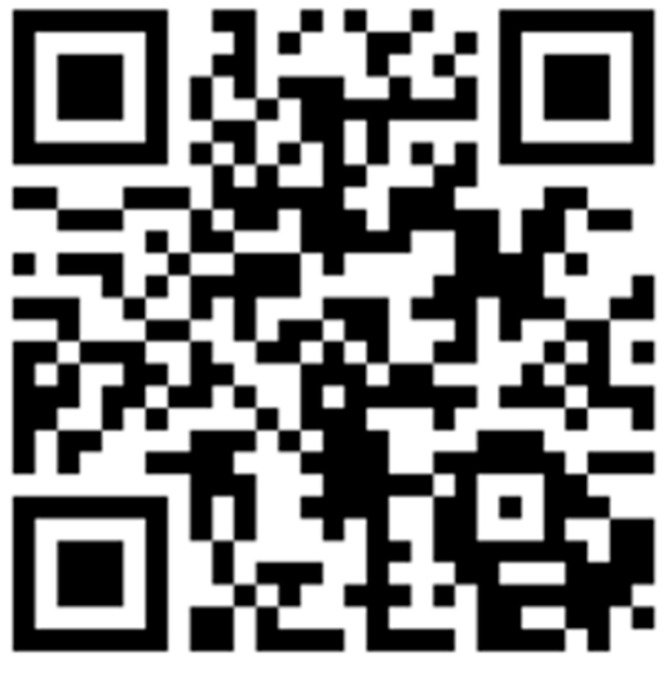
The QR code of the EFF.

Below, we report the link and the QR code for the EFF: https://forms.office.com/e/MW9M7aykWP (accessed on 15 March 2024) (see Figure 2).

## 3. Results

The results are organized into sections and subsections.

Section 3.1, “*The Outcome from the Electronic Survey*”, presents the results of administering the electronic survey to radiologic healthcare technicians. This section consists of three subsections.

Section 3.1.1, “*Insights into the Study Participants: Unveiling Characteristics of the Sample*”, characterizes the sample. 

Section 3.1.2, “*Findings from Graded, Multiple-Choice, and Likert Scale Questions*”, reports the outcome of quantitative data obtained from numerical responses (single-choice questions, multiple-choice questions, graded questions, and Likert questions with a 6-level scale). 

The last section, Section 3.1.3, “*Unveiling Insights from Open-Ended Responses: A Dual Perspective on Feedback and the Future of Home Radiology*”, reports the outcome of open-ended responses.

Section 3.2, “*The Outcome from the Electronic Feedback Form*”, presents the results of administering the CAWI to experts to gather feedback on the devised tool. It is divided into two subsections. 

Section 3.2.1, “*Identification of the Expert Observer Group*”, identifies the group of experts involved in this CAWI. 

Section 3.2.2, “*In-Depth Feedback Through the Electronic Feedback Form*”, reports the outcome of the administration of the second CAWI:

Finally, Section 3.3, “*Comprehensive Insights Summary*”, provides a synthesis of the results for the two CAWI administrations, organized into two corresponding subsections: Section 3.3.1, “*Insight summary from the Electronic survey*”, and Section 3.3.2, “*Insight summary from the Electronic Feedback Form*”.

### 3.1. The Outcome of the Electronic Survey

#### 3.1.1. Insights into the Study Participants: Unveiling Characteristics of the Sample

One significant outcome derived from this study is the development of the CAWI product, a result of careful consideration given to multiple perspectives. The individuals involved in this endeavor comprised *Bioengineers, Medical Engineers, and experts with a background in health professions and diagnostic techniques, including training in medical radiology techniques. Additionally, experts in economics and the development of Medical Devices* were part of this collaborative effort, with these first five competencies being among the authors of the work. Furthermore, contributors from the fields of *medical physics and radiological medicine* also played integral roles. Notably, no critical issues were identified across any of the submissions. It is noteworthy that the survey was completed swiftly, with participants taking an average of 79.7 s to open and complete it, never exceeding 120 s in the entire process. After the survey was opened, every participant willingly provided their responses. Notably, there are no inquiries related to cybersecurity, as the team has carefully evaluated the incorporation of the Virtual Private Network (VPN) in this context, deeming the security measures equivalent to those achievable within a local hospital setting. Consequently, the examination of cyber risks, a well-recognized concern in the hospital domain, falls outside the initial focus of this investigation.

The two tables (Table 2 and Table 3) provide details on the overall sample of interviewed Medical Radiology Technologists (MRTs) (Table 1) and the subset of those who, in some capacity, have been involved with home radiology (HR) matters (Table 2). The first table presents a comprehensive overview of the entire MRT sample interviewed, while the second table specifically focuses on those within the sample who have encountered or dealt with HR-related aspects.

#### 3.1.2. Findings from Graded, Multiple-Choice, and Likert Scale Questions

In the assessment, individually graded and Likert responses were employed, with a scale ranging from a maximum score of 5 to a minimum of 1. An average score surpassing 3.0 =$\;\frac{1+5}{2}$ signified a positive evaluation, with a higher score approaching 5 indicating a more favorable response. Conversely, a score falling below 3.0 signaled a negative evaluation, with a lower score approaching 1 indicating a more critical stance.

**Figure 3:**
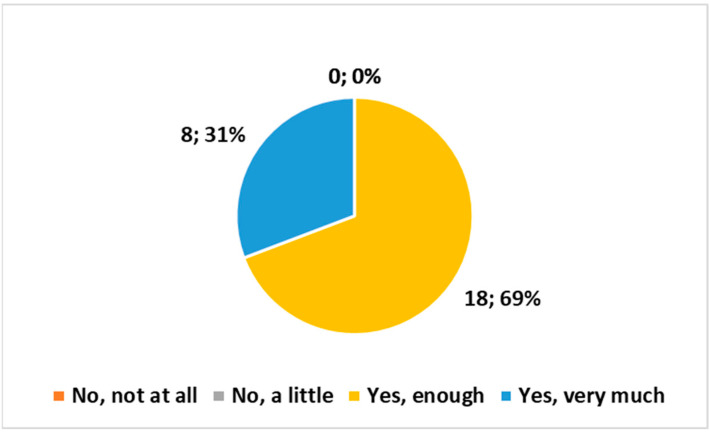
Answer to the multiple choice question, “Do you believe that the examination conducted at home complies with the safety requirements regarding exposure to ionizing radiation?”.

**Figure 4:**
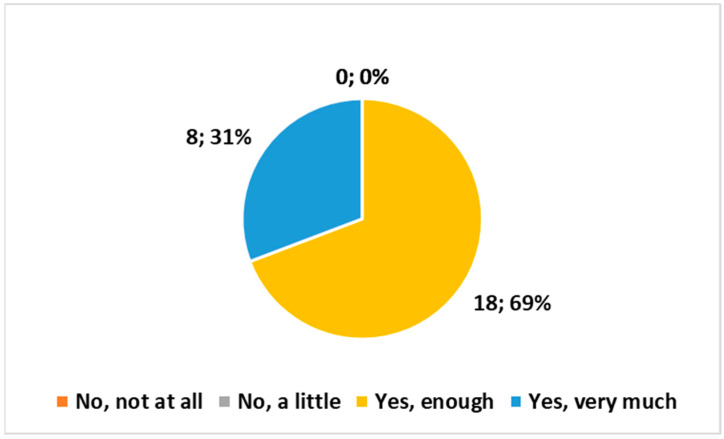
Answer to the multiple choice question, “Are the means and technologies (vehicle, PC, radiological equipment, etc.) provided by the Health Authority suitable for delivering the service?”.

**Figure 5:**
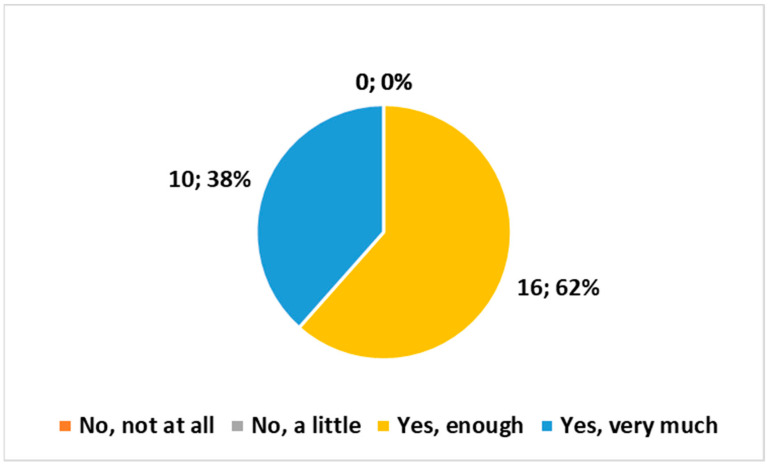
Answer to the multiple choice question, “Do you believe it is important for the MRT to be part of the Integrated Home Care team?”.

The following three multiple-choice questions (with four choices each) yielded comparable outcomes, as depicted in Figure 3, Figure 4 and Figure 5:

- *“Do you believe that the examination conducted at home complies with the safety requirements regarding exposure to ionizing radiation?”*
- *“Are the means and technologies (vehicle, PC, radiological equipment, etc.) provided by the Health Authority suitable for delivering the service?”*
- *“Do you believe it is important for the MRT to be part of the Integrated Home Care team?”*

None of the three questions received negative responses. All three exhibited a preference for the response “Yes, enough”, followed closely by “Yes very much”. The χ^2^ test indicated high significance (*p* < 0.01) in all three cases.

The graded question

- *“How important do you consider listening to the problems of the patient or family/caregivers?”*

received an average score of 4.83 (STD ± 0.21), with only 1 vote coinciding with 3 (neither positive nor negative), while all other votes were higher.

The graded question

- *“Overall, how satisfied are you with the Home Radiology service?”*

achieved an average score of 4.93 (STD ± 0.13), with all votes being ≥ 4.

**Figure 6:**
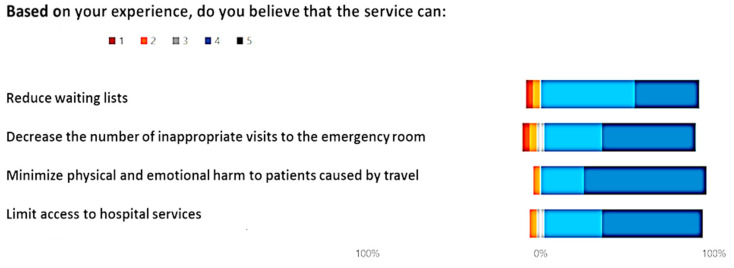
Answer to the Likert, “Based on your experience, do you believe that the service can?”.

**Figure 7:**
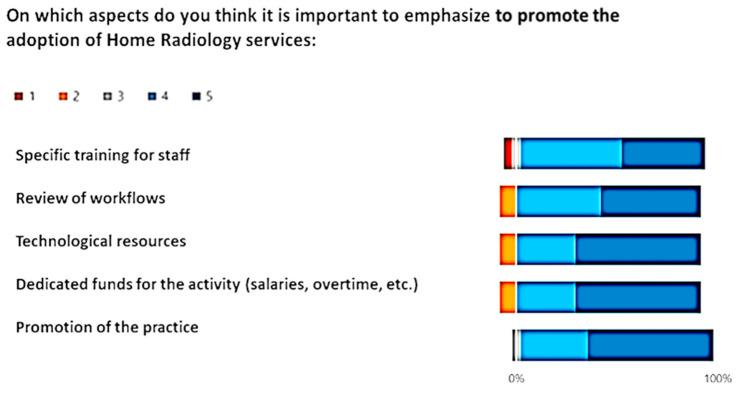
Answer to the Likert, “On which aspects do you think it is important to emphasize to promote the adoption of Home Radiology services:?”.

**Figure 8:**
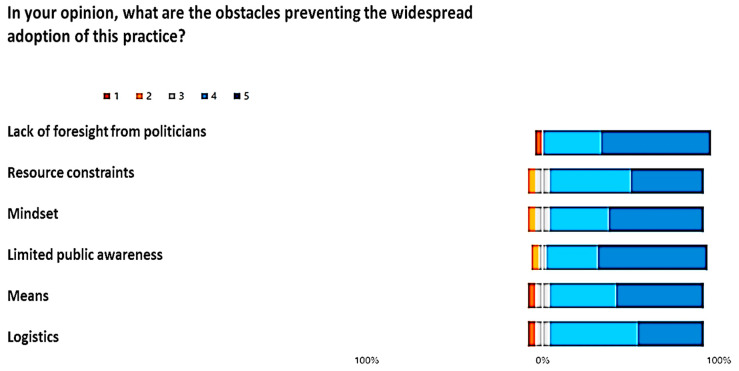
Answer to the Likert, “In your opinion, what are the obstacles preventing the widespread adoption of this practice?”.

Figure 6, Figure 7 and Figure 8 show the outcome from the three module-Likert:

Giving a comprehensive view, the Butterfly diagrams vividly highlight the overall minimal presence of the tail below 0% across all options. This observation signifies a consistently high level of positive appraisal for each presented choice. Furthermore, an approach was adopted by applying the χ^2^ test option by option, assessing the significance in the frequency of positive and non-negative ratings in comparison to negative ones. Across every option, the χ^2^ test yielded notably high significance levels (*p* < 0.01), reinforcing the statistical robustness of positive evaluations over negative counterparts.

Details:

Within the Likert scale associated with the set of options for “*Based on your experience, do you believe that the service can:*”, the most favored choice was “*Minimize physical and emotional harm to patients caused by travel*”, garnering the highest average rating of 4.3 (STD ± 0.50)

Within the Likert scale associated with the set of options for “On which aspects do you think it is important to emphasize to promote the adoption of Home Radiology services:”, the most favored choice was “Promotion of the practice”, garnering the highest average rating of 4.5 (STD ± 0.33)

Within the Likert scale associated with the set of options for ‘‘In your opinion, what are the obstacles preventing the widespread adoption of this practice?”, the most favored choice was “lack of foresight from politicians”, garnering the highest average rating of 4.4 (STD ± 0.42).

#### 3.1.3. Unveiling Insights from Open-Ended Responses: A Dual Perspective on Feedback and the Future of Home Radiology

We also present the insights derived from a global perspective through open-ended questions. In this exploration, we delve into the valuable feedback gleaned from open-ended responses, shedding light not only on the challenges and triumphs of home radiology but also on the potential applications of surveys in shaping its future landscape.

Open Question: What types of challenges have you encountered?

In the realm of home radiology, challenges manifest as nuanced facets of our commitment to providing quality healthcare. When working with individuals with significant disabilities and/or frailties, several important challenges may arise and have been reported, including communication, mobility, emotional sensitivity, accessibility, interaction with the caregiver, and cultural sensitivity. However, all those who submitted open-ended questions regarding these issues did not report any critical problems and stated that they felt prepared to face the challenge, considering it a personal reason for professional and human growth.

Open Question: What are the positive aspects that you have identified in providing the service at the patient’s home?

The provision of home radiology services brings forth a spectrum of positive aspects that profoundly impact both patients and healthcare practitioners. Conducting examinations in the familiar setting of a patient’s home, especially for those in fragile conditions, is a transformative benefit. Beyond the inherent convenience, this approach ensures a higher level of patient care by eliminating the need for them to traverse to a diagnostic center, concurrently contributing to the reduction in healthcare costs.

Moreover, the unique rapport established during home visits fosters a sense of hospitality and appreciation reminiscent of a bygone era. This not only enriches the patient’s experience but also aligns with the broader mission of combatting disability, creating a more holistic and patient-centric healthcare model.

Open Question: If you deem it appropriate, you can leave a comment on the topic of home radiology.

The comments highlight that the potential of home radiology services remains untapped without a comprehensive census, both in public and private spheres. A centralized survey is imperative to gauge the extent of utilization and, consequently, unlock the full potential of this diagnostic tool. With data-driven insights from a thorough census, home radiology can be strategically harnessed, catering to the specific needs of the healthcare landscape.

Open Question: Respecting the patient’s privacy, share an experience of home radiology that you consider significant.

As we collect data for a comprehensive report on home radiology’s contributions to the national healthcare system, the experiences gathered during the COVID-19 pandemic stand out as indelible markers. The challenges posed by the pandemic highlighted the critical role of home radiology in ensuring healthcare continuity. The stories we are assembling serve not only as a testament to the service’s importance but also as a guide for future enhancements, solidifying its role in the ever-evolving healthcare landscape.

### 3.2. The Outcome of the Electronic Feedback Form

#### 3.2.1. Identification of the Expert Observer Group

For the purposes of this investigation, we enlisted the expertise of a group consisting of 16 observers chosen for their experience in the field. They were selected based on their background in the sciences of diagnostic technical professions (training for a coordinating role in this field) and with various primary professional focuses. This deliberate and thorough selection process aimed to incorporate a diverse range of qualified perspectives, ensuring a comprehensive and well-rounded evaluation of our research.

#### 3.2.2. In-Depth Feedback through the Electronic Feedback Form

In the assessment, individually graded and Likert responses were employed, with a scale ranging from a maximum score of 6 to a minimum of 1. An average score surpassing 3.5 =$\;\frac{1+6}{2}$ signified a positive evaluation, with a higher score approaching 6 indicating a more favorable response. Conversely, a score falling below 3.5 signaled a negative evaluation, with a lower score approaching 1 indicating a more critical stance.

The response to the question “*Please indicate your level of familiarity with the topic of home radiology*” received an average rating of 5.3, with a minimum of 4 and a maximum of 6 (STD ± 0.41).

The answer to the question “*Provide your overall assessment of the proposed tool*” received an average rating of 5.1, with a minimum of 4 and a maximum of 6 (STD ± 0.52). 

**Figure 9:**
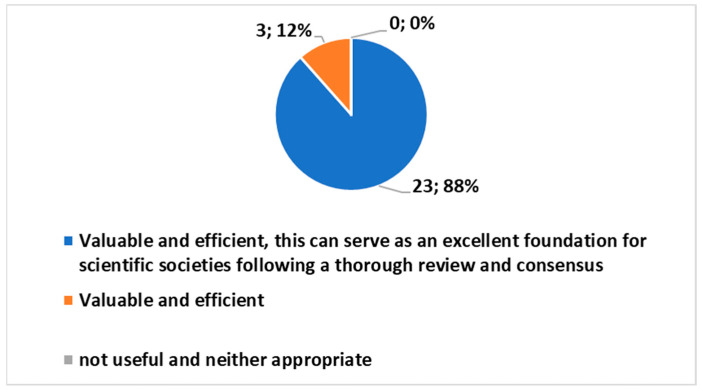
Answer to the multiple choice question, “*I think that the proposed tool is*:”.

An intriguing aspect emerges in the responses to a question offering three distinct choices, “*I think that the proposed tool is:*”. The graphical representation (Figure 9) highlights a unanimous positive sentiment toward the ES, with every option reflecting a favorable opinion. Notably, the most favored choice, selected by 88% of respondents, expressed that the survey was “Valuable and efficient, serving as an excellent foundation for scientific societies”. This overwhelming preference holds substantial significance, as demonstrated by the χ^2^ test (*p* < 0.01), underlining a robust consensus among participants regarding the commendable nature of the ES.

**Figure 10:**
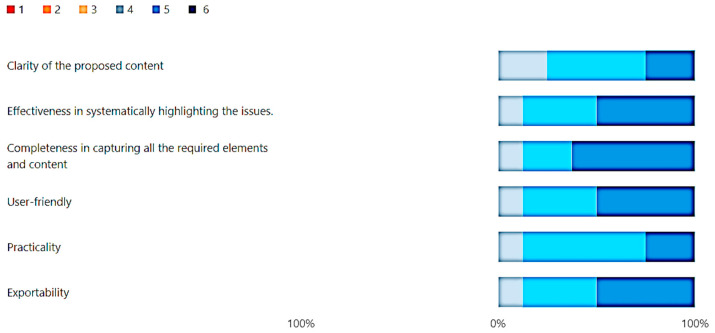
Answer to the Likert, “Provide a detailed evaluation of the following points concerning the tool”.

The Likert scale, in its findings (Figure 10), notably showcased a remarkably high level of acceptance, consistently yielding ratings never falling below 5.1 on each individual item. The Butterfly diagram further accentuates this positive trend by illustrating a complete absence of ratings below 4 percent. This absence of lower ratings obviates the necessity of applying the χ^2^ test, as it becomes apparent that the overwhelming majority of responses align positively with the subject matter, reinforcing the robust acceptance of the surveyed elements.

### 3.3. Comprehensive Insights Summary

A study with two polarities was conducted using two CAWI tools. Through the first CAWI tool, it was possible to capture feedback from medical radiology technicians familiar with the practice. The second CAWI tool allowed for obtaining feedback on the methodology used and its related perspectives.

#### 3.3.1. Insight Summary from the Electronic Survey

The assessment, utilizing graded and Likert responses on a scale of 1 to 5, unveils a favorable perspective for home radiology, where an average score exceeding 3.0 indicates positive sentiments. Multiple-choice questions consistently received affirmative responses, statistically significant at *p* < 0.01, reflecting a widespread positive perception.

Graded inquiries about the importance of listening to patient concerns and overall satisfaction garnered high average scores (4.83 and 4.93, respectively), underscoring their pivotal role. Butterfly diagrams illustrating Likert scale responses showcased a unanimous positive outlook. Specific emphasis on minimizing harm during patient travel and promoting home radiology services received high average ratings of 4.3 and 4.5.

Within the Likert scale, the question exploring obstacles to the widespread adoption of home radiology services provided valuable insights. Respondents favored the option “*lack of foresight from politicians*”, with a high average rating of 4.4, highlighting its significance in the context of adoption challenges.

Open-ended responses delved into nuanced challenges, such as physical demands and occasional biases, emphasizing the necessity for inclusivity. Conversely, positive aspects highlighted the transformative benefits of home examinations, contributing not only to patient comfort but also yielding cost reductions.

This study underscores the urgency of a comprehensive census to unlock the full potential of home radiology. Insights from the COVID-19 pandemic underscore the service’s indispensable role in maintaining healthcare continuity, offering valuable guidance for future enhancements.

In summation, the findings offer a compelling narrative of home radiology’s positive reception, supported by a blend of quantitative and qualitative assessments. These holistic insights provide a robust understanding of the service’s strengths, challenges, and avenues for continual improvement.

#### 3.3.2. Insight Summary from the Electronic Feedback Form

Engaging 16 seasoned observers with diverse expertise, our study meticulously represented crucial roles in the medical field. This deliberate selection process enriched the evaluation with a comprehensive range of qualified perspectives.

Utilizing a 1 to 6 scale, an average score above 3.5 indicated positive evaluations. The responses demonstrated a high level of familiarity with home radiology (average rating: 5.3) and a positive overall assessment of the proposed tool (average rating: 5.1). Notably, a unanimous 88% consensus favored the tool’s value and efficiency, emphasizing its excellence as a foundation for scientific societies, as confirmed by the χ^2^ test.

Consistently high ratings, never falling below 5.1, were observed across all Likert scale items. The Butterfly diagram underscored the absence of ratings below 4, affirming overwhelming positive consensus without the need for the χ^2^ test. In summary, seasoned observers validate the commendable nature of the Evaluation Survey, highlighting its efficiency and value. The consensus positions it as an excellent foundation for scientific societies, showcasing robust acceptance and positive feedback.

## 4. Discussion

### 4.1. Key Discoveries: Opportunities, Challenges, and Issues

This study delved into the pivotal role of investigating professionals’ experiences and opinions regarding home/domiciliary radiology within the health domain. A specific CAWI-based tool was employed and submitted in a peer-to-peer mode to both citizens and professionals. The focus of this work specifically concerns medical radiology technicians involved in this radiology practice. Given the inherent complexity and heterogeneity of the domains, the survey addressed various aspects. In a broader sense, this study has illuminated how an expansive electronic questionnaire within this realm can emerge as a valuable and indispensable tool. Expanding on the distinct values, this study brings forth the following results:

- *The CAWI ES Tool:*

The first contribution of this study lies in the careful design of the CAWI ES tool itself. This tool has been meticulously crafted to explore the intricacies of daily radiology practices, allowing for a detailed examination of key points and the collection of valuable feedback. Its construction ensures that it serves as a potential instrument for understanding and potentially improving the efficiency of radiological procedures in HR.

- *High Acceptance Level of the ES CAWI tool:*

Another significant aspect revealed by the study is the remarkably high acceptance level of the ES tool. This finding comes from the perspectives of a panel consisting of 16 experts who not only recognize its current utility but also see its potential as a valuable instrument in future applications. The unanimous agreement among these experts underscores the perceived credibility and effectiveness of the ES tool in the field of radiology.

- *CAWI Tools:*

Another noteworthy feature is the introduction of two CAWI tools, encompassing both the ES and the EFF. This dual-tool approach represents a substantial and final enhancement to the research methodology. Beyond their evident usefulness, these tools demonstrate a commendable level of adaptability, being easily exportable. This not only adds to the convenience of the research process but also emphasizes the practicality and versatility of the applied methodology.

- *Specific Outcome:*

This study’s last contribution is the in-depth evaluation of outcomes derived from interviews with medical radiology technicians. This thorough examination provides insights into the practical implications of the implemented methodologies and sheds light on the tangible impact on the daily practices of these healthcare professionals.

Regarding the outcome, it is essential to note that the obtained sample is not small, considering the following factors. The health domain in Italy is organized on a regional model, with the country divided into 20 regions. The use of Health Radiography (HR) varies across these regions, with some utilizing it while others do not. According to a survey [28], only four regions offered HR services in 2018. However, this landscape changed post-pandemic, with more regions, including Umbria [29], adopting this service. It is crucial to recognize that Italian regions are further divided into provinces, where HR usage may vary. For example, in Umbria (approximately 1/50 of the entire national population), HR is only used in the province of Perugia, involving an Opertavive Unit [29]. Considering these aspects, the identified sample of 26 Medical Radiology Technicians (MRTs) is entirely reasonable. An indirect suggestion to the Ministry of Health, responsible for mapping healthcare activities, is to initiate a census in this domain. A census, coupled with raising awareness through scientific societies/associations, could collectively boost both the monitoring and practice of HR. The feedback from MRTs reflects the enthusiasm and, simultaneously, significant expectations surrounding this practice, which is viewed as having promising and motivating prospects on a personal level. However, MRTs acknowledge the need for various strategic initiatives (e.g., specific personnel training, workflow revisions, technological resources, dedicated funds for activities such as salaries and overtime, and the promotion of the practice). The lack of foresight among politicians is considered an obstacle. Additionally, it is crucial to focus on the citizen and caregiver and on all the key working figures involved in this practice. Notably, medical radiologists emerge as central figures, bearing the responsibility in the medical act and playing a key role in the overall medical process. From those overseeing remote diagnostics to general practitioners managing complex eligibility identification procedures and medical physicists ensuring radiological safety, each contributes indispensably to other key individuals organizing the work, including stakeholders associated with HR practice.

It is also helpful to interpret these results in light of some historical studies in this field focused on surveying the experiences. The uniqueness and innovation of our study lie in its targeted exploration of the experiences of medical radiology technicians involved in home radiology, achieved through the application of a Computer-Assisted Web Interviewing (CAWI) survey method. From a general perspective, our approach continues that proposed by Sawyer et al. in 1995 [7] to gather feedback on this practice, but now utilizing a methodology (CAWI) that was not available in 1995. We focus on the individuals actively involved in the field, addressing new developments that have occurred over three decades. In comparison to a broader overview of surveys [11,12,13,14,15,16,17,18,19,20,21,22,23,24] that focus on teleradiology in general, of which home radiology can, in a sense, be considered an integral part, our study specifically delves into the intricacies of this particular professional group and their engagement with home radiology practices.

The other referenced overview of surveys encompasses a diverse range of investigations within the broader field of radiology but has not addressed home radiology. These include examinations of teleradiology practices in Turkey [11], perceptions of clinical medical students toward radiology careers in Ghana [12], patient-reported outcomes after fracture treatment in primary healthcare [13], usability and efficiency evaluations of an application in orthopedics [14], socio-economic and psychological impacts of the COVID-19 outbreak on radiologists [15], work-style reform and technology utilization among diagnostic radiologists in Japan [16], skepticism about artificial intelligence in the radiology field [17], patient satisfaction with teleradiology services in Italy [18], patient satisfaction with teleradiology services in general practice [19], on-call service of neurosurgeons in Germany [20], attitudes of Korean primary care family physicians toward telehealth [21], factors influencing clinician satisfaction with radiology services [22], and positive aspects found in healthcare information and communication technology implementation in Finland [23]. In contrast, our study focuses specifically on the experiences of medical radiology technicians in the context of home radiology. The use of the CAWI as a survey method provides a modern and efficient approach to gathering insights directly from this professional group, allowing for detailed feedback on their perspectives, challenges, and contributions in this evolving field. By narrowing the scope to this specific demographic, our study adds a targeted and specialized dimension to the broader landscape of radiology research [11,12,13,14,15,16,17,18,19,20,21,22,23,24]. In common with these studies, our research highlights the importance of targeted questionnaire proposals rather than standardized ones. This is evident when analyzing questionnaires proposed to investigate the introduction of innovative technologies in radiology, such as artificial intelligence. Various surveys have been proposed [30,31,32,33,34,35,36,37,38,39,40] to explore the perspectives of diverse stakeholders in this field, including radiologists, radiographers, primary care providers (PCPs), students, and patients. Research focused on patients [30,31,32] has shed light on their curiosity and general acceptance of these techniques, emphasizing the need for awareness campaigns and educational efforts and addressing cybersecurity concerns in tandem with eHealth and mHealth integration. Among students [39], prevalent curiosity and optimism were observed, but in tandem with dissatisfaction surfaced regarding the inadequacy of training, prompting a call for the integration of specific modules into their training programs. Investigations into radiologists and radiographers [34,35,36,37,38] uncovered a widespread openness to these innovative solutions. Moreover, there was a strong desire among these professionals to actively contribute to future workflow modifications, contingent upon receiving adequate training. In almost all studies, with only rare exceptions like [32], researchers opted for free and non-standardized questionnaires, employing validation processes. This implies that, in the current historical context, scholars are leveraging their creativity to construct increasingly innovative and adaptable survey instruments. Other standardized and more widely used instruments, such as the Technology Acceptance Model, have seen more limited utilization [40]. Another aspect that emerges when comparing studies conducted on teleradiology [11,12,13,14,15,16,17,18,19,20,21,22,23,24] and on the integration of artificial intelligence in radiology [30,31,32,33,34,35,36,37,38,39,40] is the need to activate national and international initiatives of this kind sponsored by societies and/or scientific federations in the field and to focus more on the entire working domain [26].

### 4.2. Takeaway Message

This study, through the application of a CAWI survey method, specifically explores the experiences of medical radiology technicians engaged in home radiology. The use of CAWI tools is highlighted as a significant innovation, providing meticulous design, high acceptance levels, and a comprehensive impact evaluation. This study’s focus on the specific professional group of MRTs, utilizing a modern approach, adds a targeted dimension to the broader landscape of HR research. The need for national and international initiatives in the field, supported by scientific societies, is emphasized to further explore the evolving landscape of the integration of HR in the health domain.

### 4.3. Work in Progress

Future work will focus on the citizen and caregiver and on all the key working figures involved in this practice, from radiologists engaged in remote diagnostics, general practitioners involved in complex eligibility identification procedures, and medical physicists ensuring radiological safety to other key individuals organizing the work, including stakeholders associated with HR practice. Concurrently, we will initiate a structured transition process with the following objectives: effectively transferring our findings and key insights to relevant scientific societies and raising awareness among key institutions regarding census initiatives. This transition is vital to ensure that the wealth of information we’ve gathered becomes an integral part of the broader scientific discourse. By fostering collaboration with scientific societies, we envision a dynamic exchange of ideas, methodologies, and best practices that will contribute to the advancement of the field. This work in progress signifies our commitment to not only conducting a comprehensive analysis but also actively participating in the knowledge-sharing ecosystem. Through this dual approach, we aspire to make meaningful contributions to both public understanding and the scientific community, fostering a continuous dialogue that propels the field of home radiology forward.

### 4.4. Key Recommendations for Advancing Further Research

We have proposed a *pilot study* that we hope will serve as a catalyst for future developments. Our envisioned direction for upcoming research recommends a focused exploration of citizens, caregivers, and key figures within the field of home radiology. Notably, medical radiologists emerge as central figures, bearing the responsibility in the medical act and playing a key role in the overall medical process. From those overseeing remote diagnostics to general practitioners managing complex eligibility identification procedures and medical physicists ensuring radiological safety, each contributes indispensably.

Additionally, we aspire for this pilot study to inspire all scientific societies of the involved professionals to continue in this direction. Among the suggestions indirectly arising is the encouragement to persist through these initiatives, concurrently working on refining and building consensus on these Computer-Assisted Web Interviewing (CAWI) tools. Simultaneously, we aim to motivate and support institutions in targeted and precise census initiatives.

## 5. Conclusions

In conclusion, this pilot study delves into the experiences and perspectives of medical radiology technicians engaged in home radiology, utilizing a CAWI survey method. The use of CAWI tools represents a significant innovation, providing a meticulously designed approach with high acceptance levels and a comprehensive impact evaluation. The study’s focused exploration of this specific professional group adds a targeted dimension to the broader landscape of HR research.

This study acknowledges the need for strategic initiatives to optimize HR integration. It suggests recommendations for advancing further research by focusing on citizens, caregivers, and key figures in home radiology. Medical radiologists are highlighted as central figures, bearing responsibility in the medical act and playing a key role in the overall medical process. From those overseeing remote diagnostics to general practitioners managing complex eligibility identification procedures and medical physicists ensuring radiological safety, each contributes indispensably. This pilot study aims to inspire scientific societies to continue in this direction, encouraging the persistence and refinement of tools. Simultaneously, census initiatives are suggested. This transition is crucial for integrating the findings into the scientific discourse and aligns with our desire for our study to actively contribute to the knowledge-sharing ecosystem.

## Figures and Tables

**Table 1:** An example of the locations/focuses of application of home radiology.

Study	Location	Focus
Andersen et al. (2023) [5]	Community settings	Implementation initiatives for patient-centered care through setting up a mobile X-ray unit in the community
Dollard et al. (2022) [6]	Residential aged care facility	Residents’ perspectives on mobile X-ray services supporting healthcare-in-place in aged care facilities
Kjelle et al. (2019) [4]	Nursing homes in Southeast Norway	Cost analysis of mobile radiography services for nursing home residents
Aldridge et al. (2015) [8]	Homeless hostels	Effectiveness of peer educators on the uptake of mobile X-ray tuberculosis screening
Kjelle and Lysdahl (2017) [9]	Nursing homes	Investigation on services in nursing homes, examining residents’ and societal outcomes
Datta et al. (2017) [10]	Public–private partnership	Detection of sputum-negative pulmonary TB through digital chest X-ray conducted via a mobile van

**Table 2:** Sample of MRTs interviewed using the CAWI ES.

Participants	Males/Females	Min Age/Max Age	Mean Age
186	80/106	34/59	45.6

**Table 3:** Subsample with experience in HR.

Experience in HR	Males/Females	Min Age/Max Age	Mean Age
26	16/10	33/58	46.3

## Data Availability

Data are contained within the article.
